# Takotsubo Cardiomyopathy Occurring Simultaneously with Acute Myocardial Infarction

**DOI:** 10.3390/life13081770

**Published:** 2023-08-18

**Authors:** Ilija Srdanović, Dragana Dabović, Vladimir Ivanović, Milenko Čanković, Teodora Pantić, Maja Stefanović, Sonja Dimić, Branislav Crnomarković, Marija Bjelobrk, Miljana Govedarica, Marija Zdravković

**Affiliations:** 1 Faculty of Medicine, University of Novi Sad, 21000 Novi Sad, Serbia; ilija.srdanovic@mf.uns.ac.rs (I.S.); vladimir.ivanovic@mf.uns.ac.rs (V.I.); milenko.cankovic@mf.uns.ac.rs (M.Č.); teodora.pantic@ikvbv.ns.ac.rs (T.P.); maja.stefanovic@mf.uns.ac.rs (M.S.); sonja.dimic@ikvbv.ns.ac.rs (S.D.); branislav.crnomarkovic@ikvbv.ns.ac.rs (B.C.); marija.bjelobrk@mf.uns.ac.rs (M.B.); miljana.govedarica.94@gmail.com (M.G.); 2 Clinic of Cardiology, Institute of Cardiovascular Diseases of Vojvodina, 21204 Sremska Kamenica, Serbia; 3 Department of Obstetrics and Gynaecology, Clinical Centre of Vojvodina, 21000 Novi Sad, Serbia; 4 University Clinical Hospital Center Bezanijska Kosa, 11000 Belgrade, Serbia; sekcija.kardioloska@gmail.com; 5 Faculty of Medicine, University of Belgrade, 11000 Belgrade, Serbia

**Keywords:** Takotsubo cardiomyopathy, acute myocardial infarction, cardiogenic shock

## Abstract

Introduction: Takotsubo cardiomyopathy (TCM) is a reversible form of cardiomyopathy characterized by transient regional systolic dysfunction of the left ventricle. Case outline: A 78-year-old woman was admitted to the general hospital due to acute inferior STEMI late presentation. Two days after admission, the patient reported intense chest pain and an ECG registered diffuse ST-segment elevation in all leads with ST-segment denivelation in aVR. The patient also showed clinical signs of cardiogenic shock and was referred to a reference institution for further evaluation. Echocardiography revealed akinesia of all medioapical segments, dynamic obstruction of the left ventricular outflow tract (LVOT), moderate mitral regurgitation, and pericardial effusion. Coronary angiography showed the suboccluded right coronary artery, and a primary percutaneous coronary intervention was performed, which involved implanting a drug-eluting stent. The patient’s condition worsened as pericardial effusion increased and led to tamponade. Pericardiocentesis was performed, resulting in the patient’s stabilization. At this point, significant gradients at the LVOT and pericardial effusion were not registered. After eight days without symptoms and stable status, the patient was discharged. Conclusions: The simultaneous presence of AMI and TCM increases the risk of developing cardiogenic shock. The cardio-circulatory profile of these patients is different from those with AMI.

## 1. Introduction

Takotsubo cardiomyopathy (TCM) is a reversible cardiomyopathy characterized by transient regional systolic dysfunction of the left ventricle [1]. It is estimated that 1–3% of all patients with acute coronary syndrome have TCM [2]. TCM is registered in 5–6% of women with acute myocardial infarction with ST elevation (STEMI) [3]. The “Fourth Definition of Myocardial Infarction” does not consider TCM as myocardial infarction [4]. Although TCM can clinically mimic myocardial infarction without obstruction of coronary arteries, it appears to be a distinctly different syndrome and should be considered separately. Today, in the era of the COVID-19 pandemic, the incidence of TCM has increased significantly and is about 7.8% [5]. The TCM mechanism is still unclear, but it is believed to be triggered by emotional or physical stress. TCM is most often described as a nonsignificant coronary artery disease (CAD). However, according to the InterTak and Mayo diagnostic criteria, significant CAD is not an exclusive factor for the development of TCM [6]. The literature describes rare cases in which acute myocardial infarction (AMI) and TCM occurred simultaneously. Acute coronary syndrome is thought to cause somatic stress and thus triggers the development of TCM [7].

In this article, we will discuss the case of a 78-year-old woman who experienced acute STEMI of the inferior region and TCM simultaneously.

## 2. Case Report

**Figure 1:**
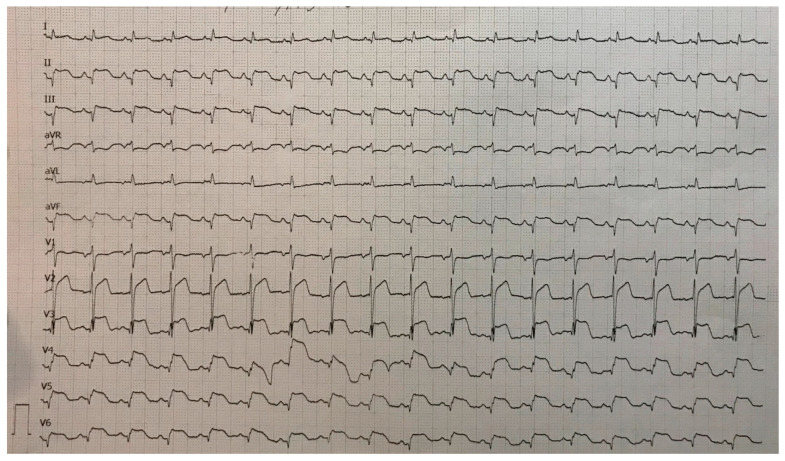
ECG registered diffuse ST-segment elevation with ST-segment denivelation in aVR and QTc interval (406 ms).

A 78-year-old woman was admitted to the general hospital due to chest pain and ECG signs of acute inferior STEMI. She had experienced chest pain 36 h prior to the admission, following a period of intense emotional stress. The patient also had anxiety as a comorbidity. Upon admission, the patient was alert and orientated, hemodynamically and rhythmically stable, and without signs of heart failure. It was concluded that the patient was a late presenter of myocardial infarction, since 36 h had passed from the beginning of the symptoms. As a result, she did not receive primary percutaneous coronary intervention, nor was she given fibrinolytic therapy. Two days after admission, the patient reported intense chest pain. The ECG registered diffuse ST-segment elevation with ST-segment denivelation in aVR and QTc interval (406 ms) (Figure 1).

**Figure 2:**
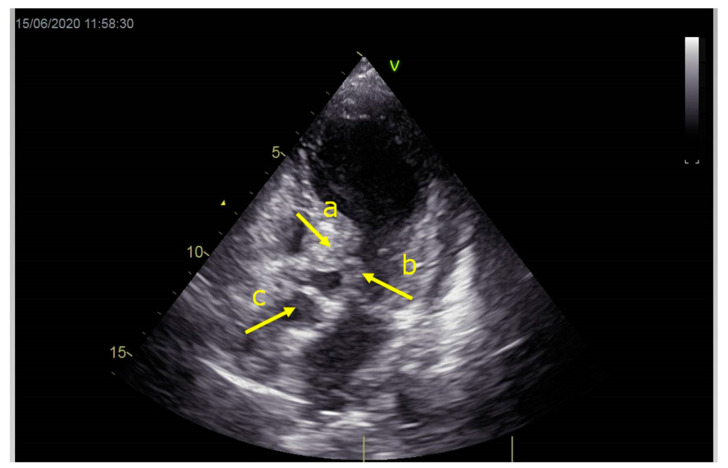
Echocardiography registered basal hyperkinetic segments of the left ventricle and formed a dynamic obstruction of the left ventricular outflow tract (a—LVOT, b—cuspi anterioris valvulae mitralis, c—aortic valve).

**Figure 3:**
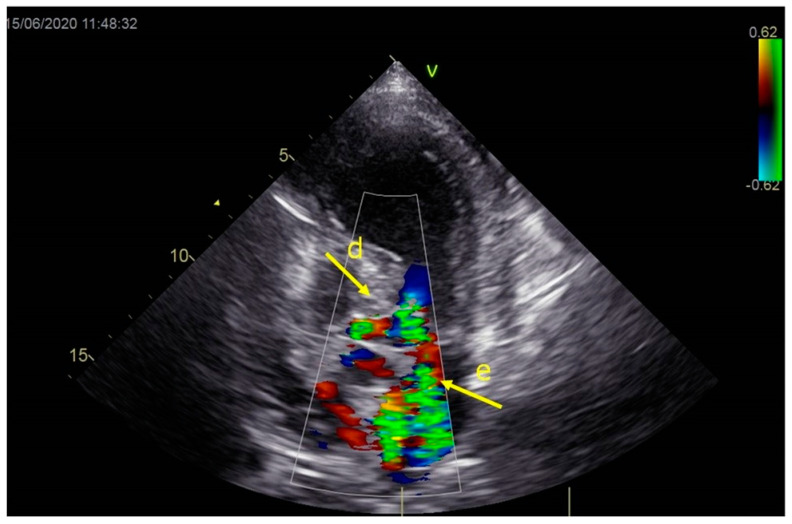
Echocardiography registered moderate mitral regurgitation (d—LVOT, e—mitral regurgitation).

**Figure 4:**
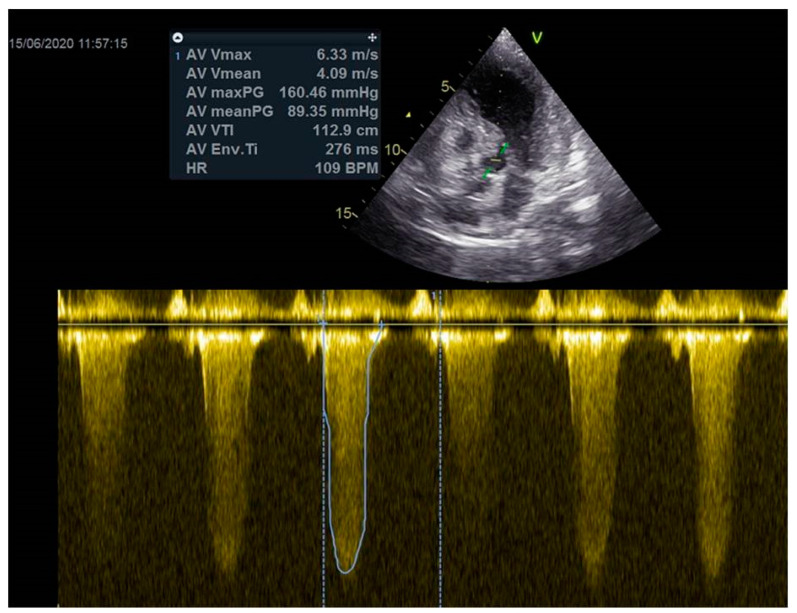
The maximum gradient above the LVOT was 160 mmHg.

**Figure 5:**
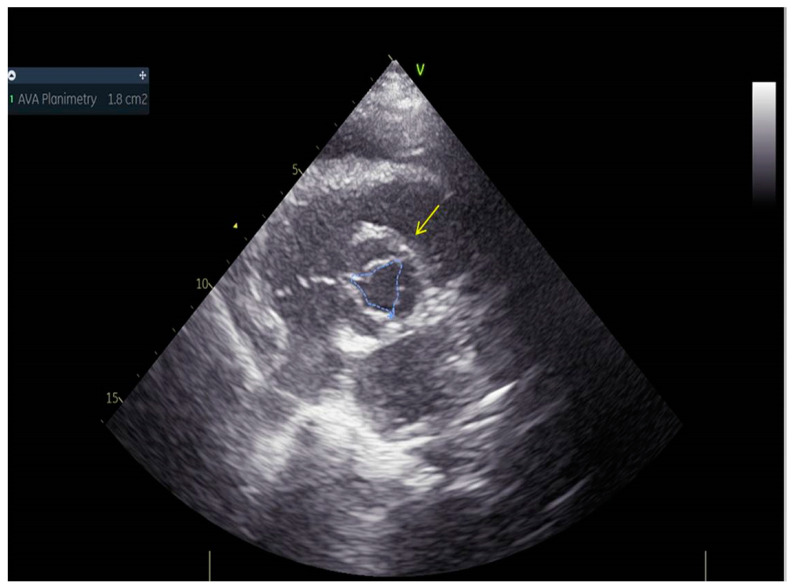
Aortic valve area was 1.8 cm^2^.

As a result, the patient was referred to a specialized medical institution. Upon admission, the patient was conscious, confused, hypotensive (TA 80/50 mmHg), with a heart rate of about 110/min, and showing clinical signs of hypoperfusion and cardiogenic shock, Killip IV. The medical staff administered sedation, inserted an endotracheal tube, and placed her on invasive mechanical ventilation. The patient was given crystalline solutions, inotrope, and vasopressor medication. An urgent echocardiographic examination was performed due to a rough systolic murmur over the precordium. It revealed akinesia of all medioapical segments of the left ventricle and akinesia basally inferior, where the myocardium was fibrously altered. Other hyperkinetic basal segments formed a dynamic obstruction of the left ventricular outflow tract (LVOTO) with turbulent flow and moderate mitral regurgitation (Figure 2 and Figure 3). The maximum gradient above the LVOT was 160 mmHg (Figure 4). The aortic valve area was 1.8 cm^2^ (Figure 5). The ejection fraction of the left ventricle (LVEF) was estimated to be 25%. The examination also revealed pericardial effusion with separation between pericardial layers along the right ventricle and atrium of up to 1.2 cm, but without any signs of tamponade.

**Figure 6:**
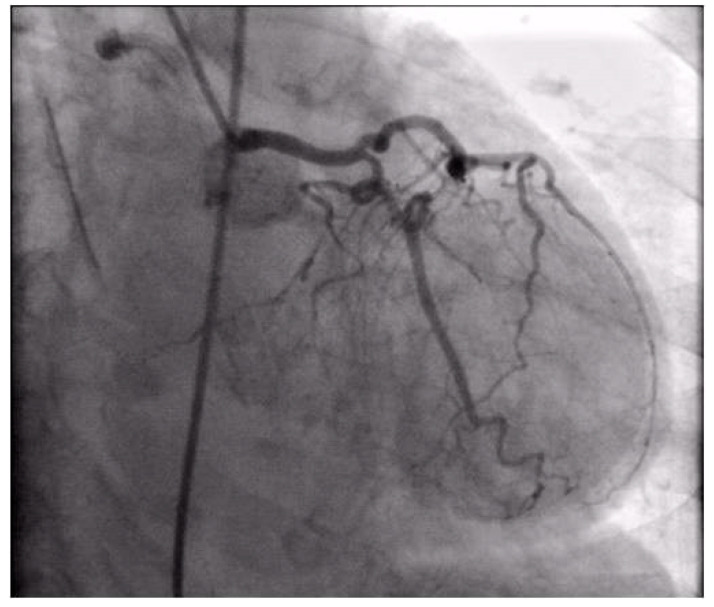
Coronary angiography registered left coronary artery without significant stenosis.

**Figure 7:**
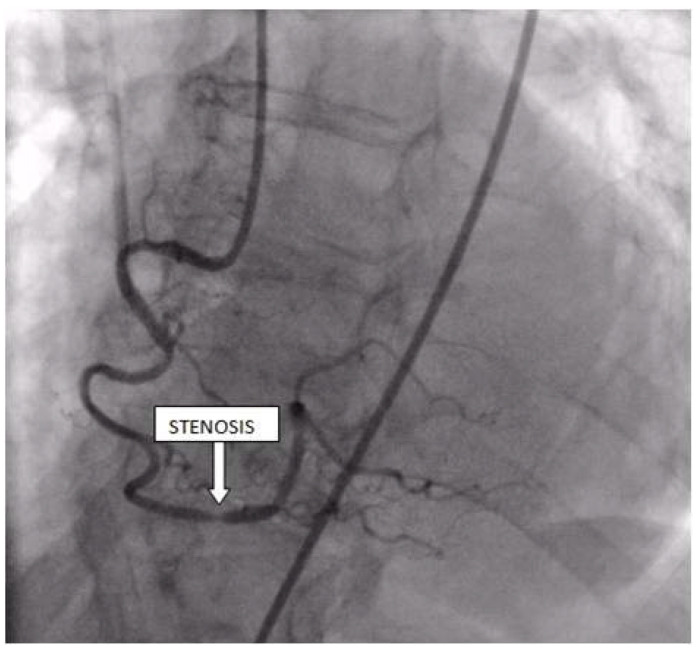
Coronary angiography registered a subocclusive lesion in the distal segment of the right coronary artery.

**Figure 8:**
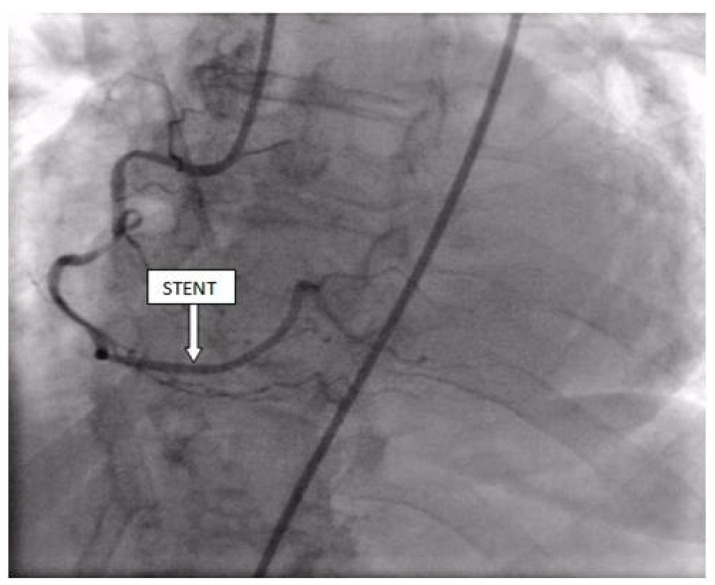
Percutaneous coronary intervention was performed, and a drug-eluting stent was implanted in the RCA, with the optimal result.

Coronary angiography was performed, showing the left coronary artery (LCA) was without significant lesions (Figure 6). On the right coronary artery (RCA), a subocclusive lesion was registered in the distal segment (Figure 7). Initially, it was thought to be a spasm, but the lesion persisted even after administering nitroglycerin intracoronary. Therefore, a primary percutaneous coronary intervention (pPCI) was performed with the implantation of a drug-eluting stent 16 × 25 mm (Boston Scientific, Marlborough, MA, USA) in the RCA, achieving the optimal result of the intervention (Figure 8).

**Figure 9:**
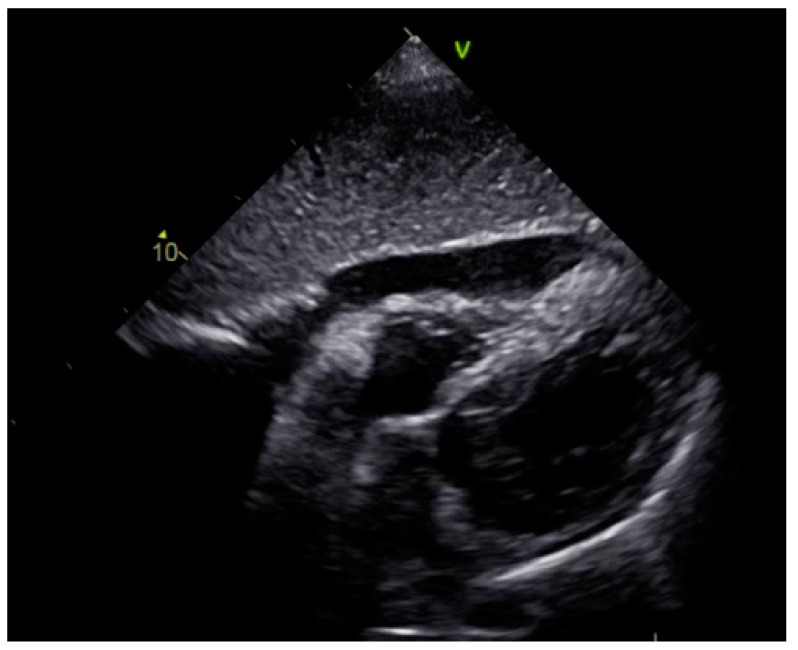
Echocardiography registered a larger amount of effusion around the heart compared to the first exam, with signs of cardiac tamponade.

**Figure 10:**
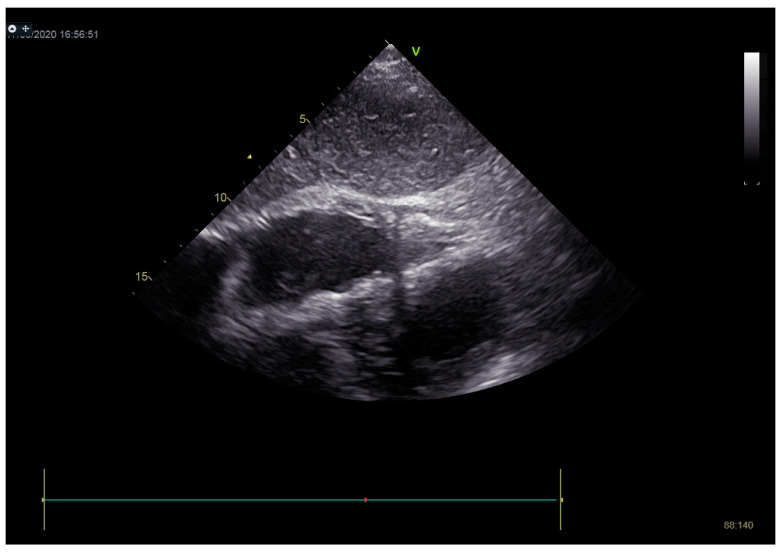
On echocardiography after pericardiocentesis, pericardial effusion was not registered.

In the following course of treatment, the patient became hemodynamically unstable, despite high doses of inotropes and vasopressors. Echocardiography registered a more significant amount of effusion around the heart compared to the previous exam, with signs of cardiac tamponade (Figure 9). Pericardiocentesis was performed, and 260 mL of hemorrhagic fluid was drained (Figure 10).

**Figure 11:**
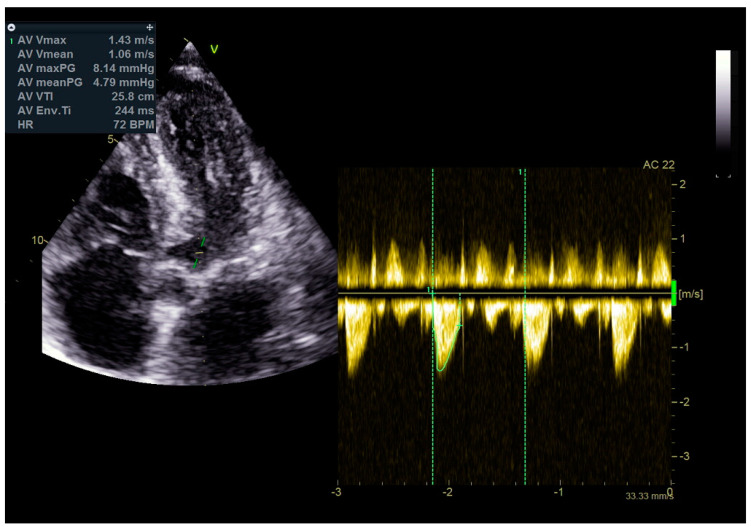
No significant gradients were registered above the LVOT, measured by CW Doppler echocardiography.

**Figure 12:**
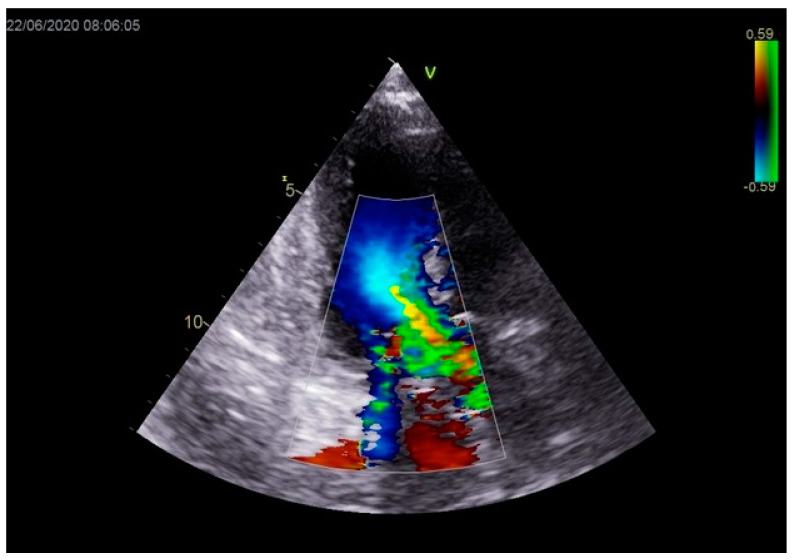
Mild mitral regurgitation was registered with color Doppler echocardiography.

Stabilization was achieved gradually, and vasopressor and inotrope support were excluded. By the second day of hospitalization, sedation was stopped, and the patient was alert and responsive. Invasive mechanical ventilation was no longer necessary, and the patient was successfully extubated. On the seventh day of hospitalization, a control echocardiographic examination registered inferior wall akinesia and hypokinesia apically, anteroseptally, inferoseptally, anteriorly, and inferiorly with an estimated LVEF of 52%. No significant gradients were found above the LVOT and there was no pericardial effusion (Figure 11). Mild mitral regurgitation was also registered (Figure 12), but there was no systolic murmur over the precordium.

**Figure 13:**
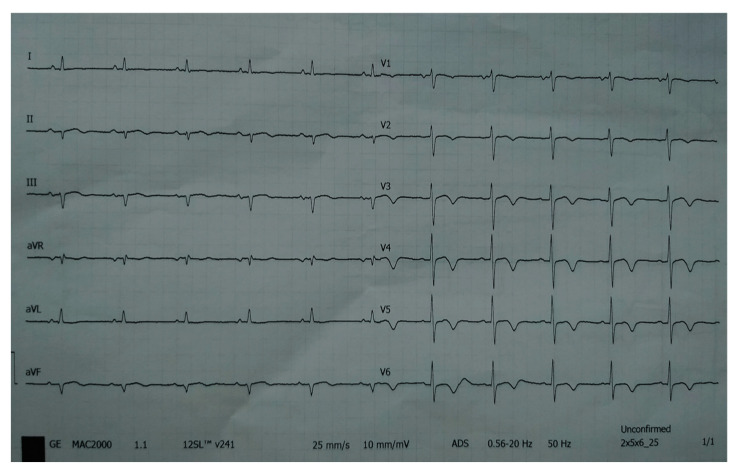
ECG registered negative T-wave in anterior leads.

On the eighth hospital day, the patient was asymptomatic, hemodynamically and rhythmically stable, and without signs of heart failure. The ECG showed a negative T-wave in the anterior leads (Figure 13). She was discharged with acetylsalicylic acid, Ticagrelor, Bisoprolol, Ramipril, a statin, and a proton pump inhibitor.

At the one-year follow-up examination, the patient was asymptomatic.

**Figure 14:**
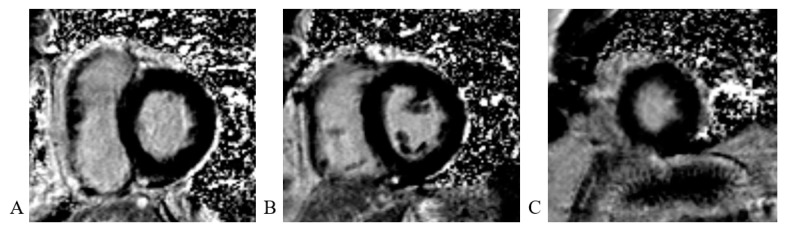
Cardiac magnetic resonance imaging: (**A**) LGE PSIR sequence, short axis, basal view; without visible zones of LGE phenomenon; (**B**) LGE PSIR sequence, short axis, mid chamber view; without visible zones of LGE phenomenon; (**C**) LGE PSIR sequence, short axis, apical view; without visible zones of LGE phenomenon.

Cardiac magnetic resonance (CMR) imaging performed per protocol showed no visible zones of late pericardial enhancement (LGE) phenomenon (Figure 14).

**Figure 15:**
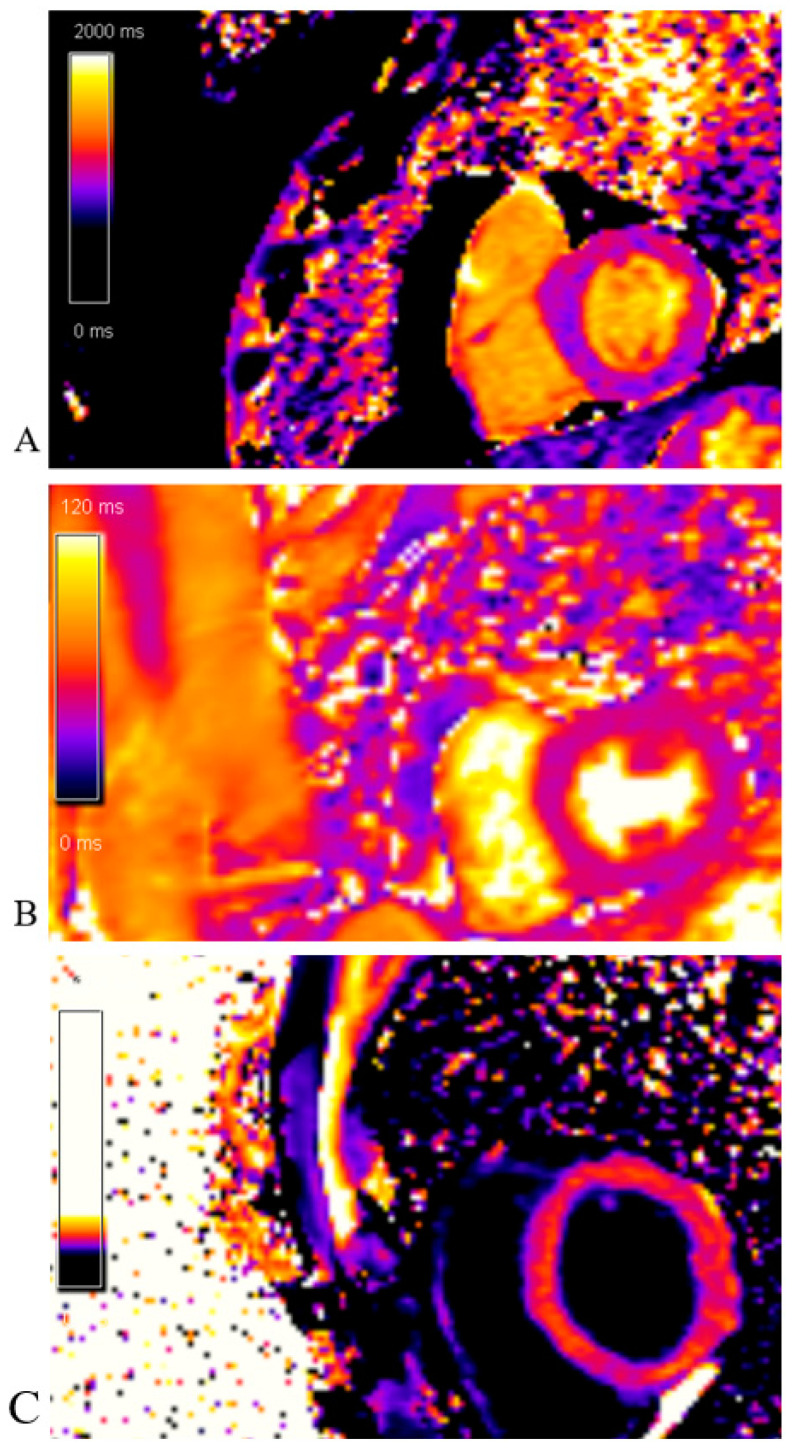
Cardiac magnetic resonance imaging: (**A**)Native T1 mapping sequence; without areas of prolonged native T1 time (edema/fibrosis); (**B**) T2 mapping sequence; without areas of prolonged T2 time (edema); (**C**) Post-contrast T1 mapping sequence; without areas of shortened post-contrast T1 time (fibrosis).

The native T1 mapping sequence was without areas of prolonged native T1 time (edema/fibrosis). The post-contrast T1 mapping sequence was without areas of shortened post-contrast T1 time (fibrosis) as well (Figure 15).

A written consent to publish this report was obtained from the patient.

## 3. Discussion

The mechanism of TCM is not yet fully understood, but it is believed to be triggered by emotional or physical stress. Emotional stress is the cause in 20–39% of cases, while somatic stress is responsible in 35–55% [8]. There are two important aspects of physiology to take into account. The first is the cognitive centers of the brain and hypothalamic–pituitary–adrenal axis, which plays a role in how stress is perceived and how much epinephrine and norepinephrine are released in response to stress [9]. The second is the response of the cardiovascular and sympathetic nervous systems to the sudden sympathetic activation and a surge in circulating catecholamines [9]. It is stated in the literature that the increased concentration of catecholamines caused by emotional or physical stress causes coronary vasospasm and microcirculation abnormalities, which may be one of the explanations for the development of TCM [10,11]. However, the SMINC-2 trial, which included patients with Takostubo cardiomyopathy, showed no evidence of massive catecholamine elevations [12]. Somatic stressors include intracranial events, severe infections, and surgical trauma [13]. The literature has also reported cases of elderly patients experiencing both AMI and TCM simultaneously [1]. AMI, as an intense somatic stressor, is thought to contribute to increased catecholamine concentrations and thus is a trigger for the development of TCM. Also, there is a significantly elevated concentration of catecholamines in the peri-infarction zone, and simultaneous occurrence of AMI and TCM can be expected.

TCM is most often described with nonsignificant CAD. However, according to the Mayo and InterTak diagnostic criteria, significant CAD is not an exclusive criterion for TCM [14,15]. In a study involving 413 patients admitted to the intensive care unit due to acute coronary syndrome, 5 patients also had CAD and TCM simultaneously [16]. This conclusion was reached after reviewing echocardiographic findings retrospectively. The simultaneous presence of CAD and TCM is associated with a higher risk of developing cardiogenic shock, the need for invasive mechanical ventilation, and the occurrence of death of any etiology. Our patient also developed cardiogenic shock, followed by the necessary involvement of invasive mechanical ventilation.

Diagnosing TCM is often a challenge. Echocardiographic examination with coronary angiography and ventriculography is the gold standard in the diagnosis. Careful examination of the coronary anatomy from several angiographic sections is necessary. Transthoracic echocardiographic examination is the first line in the diagnosis. Echocardiographic parameters indicating a high risk of TCM are low minute volume, LVEF below 35%, diastolic dysfunction, LVOTO, mitral regurgitation, right ventricular involvement, left ventricular thrombus, pericardial effusion, and rupture of the free wall [17]. Most of the listed parameters were registered in our patient upon admission.

CMR is useful for differential diagnoses. Typically, patients with stress cardiomyopathy do not present significant late enhancement, while subendocardial late enhancement is common in myocardial infarction, and focal or subepicardial late enhancement is frequent in myocarditis [18]. Our patient did not exhibit any late enhancement.

According to our findings, the evidence of Takotsubo cardiomyopathy (TCM) was transient akinesia of all medioapical segments of the left ventricle and hyperkinetic basal segments that were not registered on control echocardiography. Per the consensus document, the apical ballooning type was known as the typical TCM form, which occurs in most cases [14].

Pericardial effusion in our patient was most likely a consequence of bleeding per diapedesis since the effusion was hemorrhagic and the patient was a late presenter of myocardial infarction.

An InterTac score has been developed to assess the likelihood of TCM. It consists of seven variables: female sex (25 points), emotional stress (24 points), physical stress (13 points), absence of ST-segment depression (12 points), psychiatric disorders (11 points), neurological disorders (9 points), and prolonged QTc interval (6 points) [19]. When the patient’s score is more than 70, the probability of TCM is over 95%. In our patient, the value of InterTak was almost 72 (female sex, emotional stress, no ST segment depression, psychiatric disorders).

There are still no results from a randomized clinical study on the prognostic significance of any medication. Given the possibility that the toxic effect of catecholamines may cause TCM, the use of beta-blockers should be considered. ACE inhibitors also play a significant role in long-term therapy. On the other hand, about 20% of patients with TCM present with LVOTO and cardiogenic shock. The cardio-circulatory profile of these patients is different from those with AMI and cardiogenic shock. Low peripheral resistance and low blood pressure with poor tissue perfusion are registered. In these patients, levosimendan and mechanical circulatory support should be preferred, while inotropes and vasopressors should be avoided [20]. In our case, stabilization was rapidly achieved after the pPCI and pericardiocentesis, and inotropes and vasopressors were quickly stopped. Although levosimendan was not available at the time, mechanical circulatory support was considered, but ultimately not necessary due to the fast stabilization. It is important to note that while a beta-blocker may help reduce obstruction of LVOT, it should not be used in severe acute heart failure and hypotension.

Today, in the era of the COVID-19 pandemic, a large percentage of patients with COVID-19 infection have complications in the cardiovascular system. The most common are acute myocarditis, myocardial infarction, arrhythmias, and pulmonary thromboembolism. However, cases of COVID-19 infection complicated by TCM have also been described in the literature [21]. On the other hand, being afraid of COVID-19 infection, the patients contact the medical service later, and there is a significantly higher percentage of patients with AMI who appear as late presenters of the disease, as in the case of our patient [22]. Patients must be educated about the importance of immediately contacting an emergency department if they experience chest pain. It is crucial because the success of pPCI in late presenters is limited.

The prognosis of patients with TCM significantly depends on the presence of CAD. According to data from the Swedish Registry for Coronary Angiography and Angioplasty from the period from 2009 to2013, mortality is substantially higher in patients with TCM and CAD [3]. After one year, our patient is asymptomatic, hemodynamically and rhythmically stable, and without signs of heart failure.

In conclusion, the simultaneous presence of AMI and TCM is associated with a higher risk of developing cardiogenic shock. Patients with TCM and AMI have a unique cardio-circulatory profile compared to those with only AMI. They often present with low blood pressure, poor tissue perfusion, and low peripheral resistance. Treating these patients is challenging, as limited research is available, and each case must be approached individually. Treatment options such as levosimendan and mechanical circulatory support are recommended, while inotropes and vasopressors should be avoided.

## Data Availability

No new data were created or analyzed in this study. Data sharing is not applicable to this article.
